# “Super p53” Mice Display Retinal Astroglial Changes

**DOI:** 10.1371/journal.pone.0065446

**Published:** 2013-06-07

**Authors:** Juan J. Salazar, Roberto Gallego-Pinazo, Rosa de Hoz, Maria D. Pinazo-Durán, Blanca Rojas, Ana I. Ramírez, Manuel Serrano, José M. Ramírez

**Affiliations:** 1 Instituto de Investigaciones Oftalmológicas “Ramón Castroviejo”, Universidad Complutense de Madrid, Madrid, Spain; 2 Facultad de Óptica y Optometría, Universidad Complutense de Madrid, Madrid, Spain; 3 Facultad de Medicina, Universidad Complutense de Madrid, Madrid, Spain; 4 Spanish National Cancer Research Center, Madrid, Spain; 5 Ophthalmology Department of the University and Polytechnic Hospital La Fe, Valencia, Spain; 6 Ophthalmic Research Unit “Santiago Grisolia” Faculty of Medicine, University of Valencia, Valencia, Spain; Dalhousie University, Canada

## Abstract

Tumour-suppressor genes, such as the p53 gene, produce proteins that inhibit cell division under adverse conditions, as in the case of DNA damage, radiation, hypoxia, or oxidative stress (OS). The p53 gene can arrest proliferation and trigger death by apoptosis subsequent to several factors. In astrocytes, p53 promotes cell-cycle arrest and is involved in oxidative stress-mediated astrocyte cell death. Increasingly, astrocytic p53 is proving fundamental in orchestrating neurodegenerative disease pathogenesis. In terms of ocular disease, p53 may play a role in hypoxia due to ischaemia and may be involved in the retinal response to oxidative stress (OS). We studied the influence of the p53 gene in the structural and quantitative characteristics of astrocytes in the retina. Adult mice of the C57BL/6 strain (12 months old) were distributed into two groups: 1) mice with two extra copies of p53 (“super p53”; n = 6) and 2) wild-type p53 age-matched control, as the control group (WT; n = 6). Retinas from each group were immunohistochemically processed to locate the glial fibrillary acidic protein (GFAP). GFAP+ astrocytes were manually counted and the mean area occupied for one astrocyte was quantified. Retinal-astrocyte distribution followed established patterns; however, morphological changes were seen through the retinas in relation to p53 availability. The mean GFAP+ area occupied by one astrocyte in “super p53” eyes was significantly higher (p<0.05; Student’s t-test) than in the WT. In addition, astroglial density was significantly higher in the “super p53” retinas than in the WT ones, both in the whole-retina (p<0,01 Student’s t-test) and in the intermediate and peripheral concentric areas of the retina (p<0.05 Student’s t-test). This fact might improve the resistance of the retinal cells against OS and its downstream signalling pathways**.**

## Introduction

The p53 tumour-suppressor gene is expressed ubiquitously in all cell types as an inactive, latent transcription factor that becomes active only when the cells are subjected to a variety of cellular insults such as DNA damage, radiation, hypoxia, telomere erosion, nutrient deprivation, transcription inhibition, depletion of nucleotide pools, oncogene expression, heat shock, or oxidative stress (OS), among others [1]–[6]. The activation of p53 triggers a complex transcriptional program that, depending on the cell type, environment, and other contributing factors, induces a number of different responses, ranging from the induction of cell-cycle arrest, programmed cell death, and senescence, to DNA repair, control of mitochondrial respiration, and angiogenesis inhibition [7]–[9].

Recently, an evolving concept in cell and molecular neuroscience is that glial cells are far more fundamental to disease progression than previously thought, possibly through a noncell-autonomous mechanism that is heavily dependent on p53 activities [10]. In astrocytes, p53 promotes cell-cycle arrest by repressing c-myc transcription and/or by activating the cyclin-dependent kinase inhibitor p21cip/Cdkn1a [11]–[14]. Increasingly, astrocytic p53 is proving fundamental in orchestrating neurodegenerative disease pathogenesis. It has been reported that NMDA-mediated CNS excitotoxicity generates a hypertrophic astrocyte morphology associated with changes in p53 expression and nuclear active caspase-3 in the absence of cell death [15]. In addition, p53 is involved in oxidative stress-mediated astrocyte death after stimulation by the intercellular messenger nitric oxide (NO) [16] and by direct, transcription-independent signalling to the mitochondria [17].

Under normal physiological conditions, p53 may help to lower intracellular reactive oxidative species (ROS) levels by promoting glutathione-dependent ROS scavenging [18], [19]. In terms of ocular disease, p53 may play a role in hypoxia due to ischaemia [20], leads to G1 arrest upon retinal exposure to ionizing radiation [21]–[23], and is involved in the retinal response to OS, since p53 can stimulate the expression of specific genes that minimize or block OS [18], [19], [24]–[26].

The retina is particularly sensitive to OS because of its oxygen- and lipid-rich environment [27]–[29]. The OS and its downstream signalling pathways have been related to the pathogenesis of potentially blinding ocular diseases, including glaucoma, diabetic retinopathy and age-related macular degeneration (ARMD) [30]–[34]. There is substantial evidence that astrocytes have key functions in antioxidant processes [35] because they possess high concentrations of antioxidant enzymes (vitamin E, ascorbate, glutathione). This constitutive expression of antioxidants indicates that astrocytes may take part in the early detoxification of ROS, before inducible scavengers are synthesised [36].

Garcia-Cao et al. (2002) generated “super p53” mice carrying supernumerary fully functional copies of the p53 gene in the form of large genomic transgenes. These super p53 mice were significantly protected from cancer when compared with normal mice and showed no indication of premature ageing. This latter finding is the result of the normal regulation of the supernumerary p53 gene. Because of this, basal levels of p53 activity remained unaltered [37].

In the present study, we take advantage of this experimental model to further challenge the role of p53 in retinal macroglia. For this, we study qualitative and quantitative changes in the astrocyte populations of the super p53 mice retinas, as compared to the WT ones.

## Materials and Methods

### 1. Ethics Statement

Mice were treated in accordance with the Spanish Laws and the Guidelines for Humane Endpoints for Animals Used in Biomedical Research. The Spanish National Cancer Research Centre (CNIO) is part of the “Carlos III” Health Institute (ISCIII) and all protocols were previously submitted to and approved by the Ethics Committee of the ISCIII; approval ID numbers: PA-45 v2, PA-312, and PA-130/07. Also, animal manipulations followed institutional guidelines, European Union regulations for the use of animals in research, and the ARVO (Association for Research in Vision and Ophthalmology) statement for the use of animals in ophthalmic and vision research.

### 2. Animals and Anaesthetics

Two groups of mice of the C57BL/6 strain, aged 12 months, were considered: i) genetically manipulated mice by introducing two extra copies of p53 gene [37] (“super p53”; n = 6) and ii) wild-type age-matched control (WT; n = 6). The generation and genotyping of transgenic mice has been previously described [37]. All animals were housed in cages, maintained in temperature- and light-controlled rooms with a 12-h light/dark cycle. Animals had *ad libitum* access to food and water. Light intensity within the cages ranged from 9 to 24 luxes. Mice were maintained under constant conditions for at least 7 days prior to the experiments, which were conducted in the tumour-suppression laboratory (National Oncology Research Centre, Madrid, Spain).

Animals from both groups were examined for morphological characteristics and weighed, and all data were recorded. The mice were deeply anesthetized with an intraperitoneal (i.p.) injection of a mixture of Ketamine (75 mg/kg, Ketolar®, Parke-Davies, S.L., Barcelona, Spain) and Xylazine (10 mg/kg, Rompún®, Bayer, S.A., Barcelona, Spain) and were perfused transcardially through the ascending aorta first with saline and then with 4% paraformaldehyde in 0.1 M phosphate buffer (PB) (pH 7.4). The eyes were post-fixed for 4 h in the same fixative (4% paraformaldehyde in 0.1 M PB at pH 7.4) and kept in sterile 0.1 M PB. The retinas from both groups were dissected and processed as retinal whole-mounts and used for immunohistochemical techniques [38].

### 3. Immunohistochemistry

#### 3.1. Staining procedure

The mice retinas were immunostained as described elsewhere [39] with anti-GFAP (GFAP clone GA-5; Sigma, USA) in a 1/150 dilution. Binding sites of the primary antibody were visualized after two days of incubation with the corresponding secondary antibody: the immunoglobulin fraction of goat antimouse antibody conjugated to fluorescein isothiocyanate (FICT) (Sigma, Saint Louis, Missouri, USA) diluted 1/100. A negative control was performed to demonstrate that the secondary antibody reacted only with their respective primary antibody. This control was made by eliminating primary antibody and replacing it with antibody diluent. In addition to identifying the contribution of the endogenous fluorescence to the observed label, a sample of tissue was incubated in all the buffers and detergents used in the experiment but without antibodies.

#### 3.2. Retinal analysis and astrocyte counting

Mice retinal whole-mounts were examined and photographed with a fluorescence microscope (Zeiss, Axioplan 2 Imaging Microscope) equipped with appropriate filter for fluorescence-emission spectra of fluorescein isothiocyanate (Filter set 10, Zeiss).

Retinal astrocytes were quantified following a masked procedure. Quantification was made in the retinal whole-mount as follows. Each entire retinal whole-mount was analysed using the motorized stage of the microscope to scan the whole preparation along the x-y-z axis. Thus, all subsequent fields analysed were contiguous and were examined systematically to ensure that no portion of the retinal whole-mount would be omitted or duplicated. Photographs of these fields were taken at 20×, providing an area of 0.18890 mm^2^ and GFAP(+) astrocytes were manually counted in each photograph using the manual counting tool of the Metamorph Imaging System.

**Figure 1:**
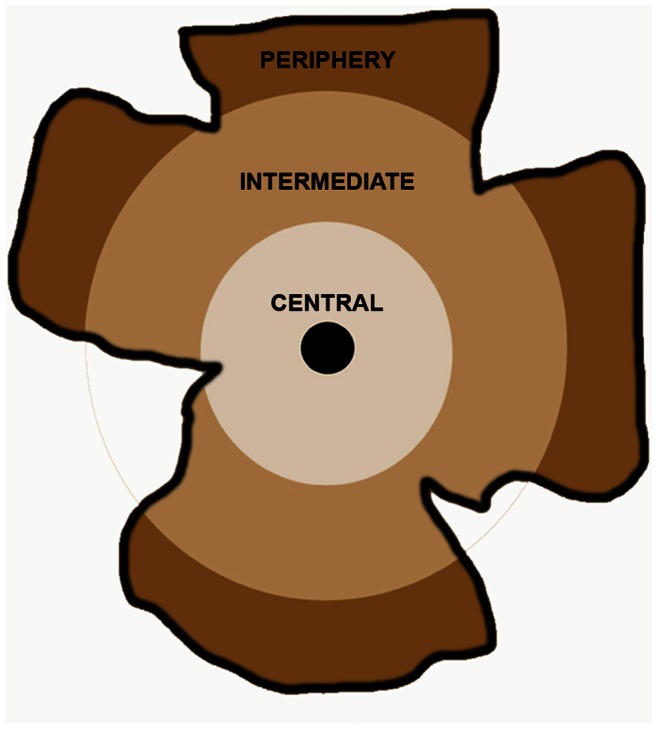
Division of the retina in concentric zones for study.

For the study of astrocyte distribution each retinal whole mount was divided into three zones that extended concentrically from the optic nerve to the periphery as follows: central (zone 1), intermediate (zone 2), and peripheral (zone 3). Equivalent areas of the retina were consistently selected for each retinal whole-mount, which included zone 1, 2 and 3 (Fig. 1).

To analyse the area occupied for each astrocyte, we used a computer-assisted morphometric analysis system (Metamorph Imaging System, version 5; Universal Imaging Corp., Downingtown, PA, USA) in association with an imaging microscope (Axioplan 2; Zeiss, Göttingen, Germany). Ten to twelve photomicrographs from each animal were taken at random from each retina. The only selection criteria were good tissue quality, good staining, clear visualization of astrocytes, and no GFAP+ Müller cells. Photographs were taken at 20×, covering an area of 0.18890 mm^2^. The resulting images were processed first with the detect edges command and then with the auto threshold command of the computer-assisted morphometric analysis system (Metamorph Imaging System, version 5; Universal Imaging Corp). The “detect edges” command isolates and enhances the edges in an image by using a selected edge-detection convolution which detects edges in the image by comparing brightness changes in the neighbouring pixels. The thresholding command defines a range of gray-scale values found on the pixels of objects of interest, differentiating them from other parts of the image based on the images’ gray scale. Areas of the image that were marked with the red threshold overlay as a visual indicator of the thresholded areas were included in the measurement and processing [40]–[42]. Thus, in each photograph astrocytes were: i) first, outlined with the detect-edges command and; ii) second, marked with a red threshold overlay (as a visual indicator of the threshold areas) for automatic calculation of the area occupied by GFAP+ astrocytes; iii) third, manually counted. To determine the mean GFAP+ area occupied by one astrocyte, the GFAP+ area of each picture was divided by the number of thresholded astrocytes in that picture.

### 4. Statistical Procedures

The statistical data were entered on a spread sheet (Excel Microsoft Co, Redmond, WA, USA) and descriptive statistics (mean ± SDM in the figure) were calculated. Differences between groups were evaluated and processed in a SPSS 19.0 (comprehensive statistical software, SPSS sciences Inc©, Chicago, IL, USA). Significance levels were set at P<0.05 (*), P<0.01 (**).

The unpaired Student’s t-test was used to compare: i) astrocyte number between WT and “super p53” mice retinas; ii) astrocyte number among the three concentric zones of the retina selected for study (central, intermediate, and peripheral); iii) mean GFAP+ area occupied by one astrocyte between WT and “super p53”.

## Results

The mice from the two groups showed no statistically significant changes in the body weight, size, eyes, or ocular adnexa tissues examined.

### 1. GFAP Staining

#### 1.1. wild-type C57BL/6 mice

**Figure 2:**
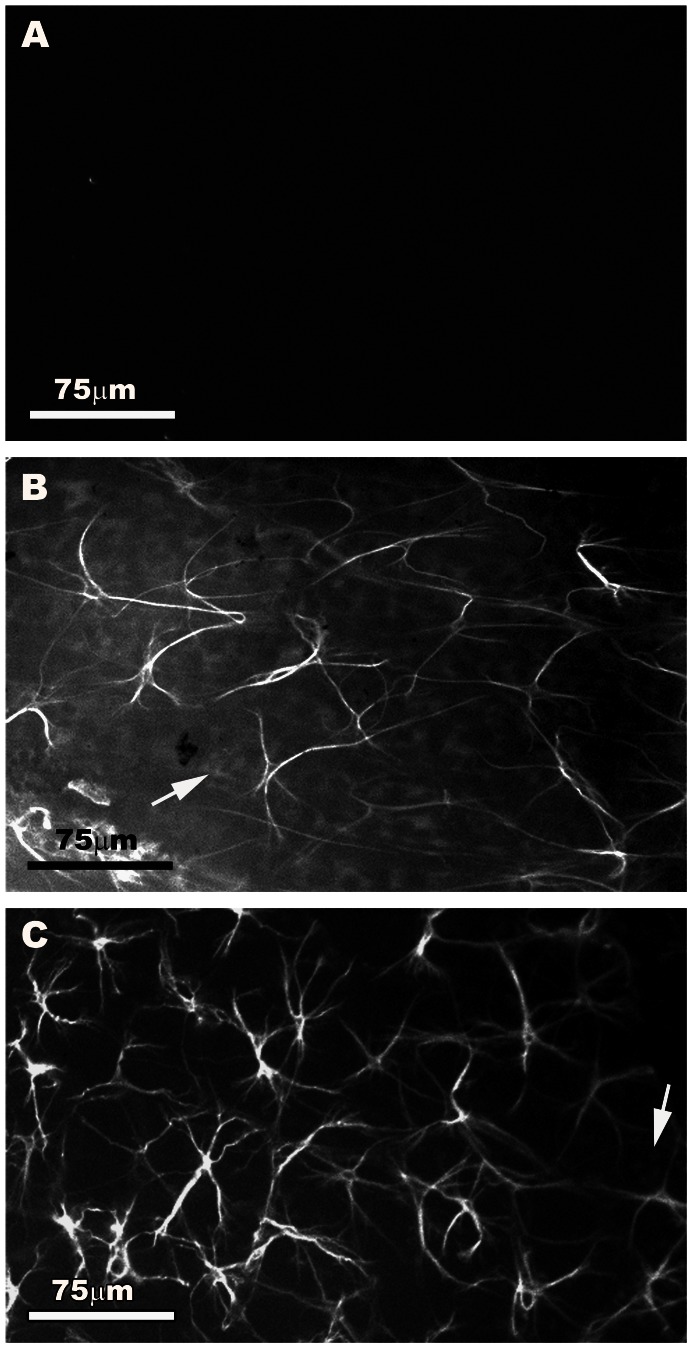
Astroglial plexus of equivalent areas (central zone) of the retinal whole-mounts in WT and “super p53” mice. GFAP immunofluorescence. A: negative control for GFAP immunostaining, B: WT. C: “super p53”. In both B and C, Müller cells appeared as faint GFAP+ punctated structures between astrocytes in some retinal areas (arrow) and astrocytes formed a homogeneous plexus on the nerve-fiber-RCG layer of GFAP+ cells regularly distributed throughout the retina. This plexus was composed of stellate cells that could easily be distinguished from each other. In “super p53” the astrocyte plexus was denser than in WT. [WT: wild type p53 age-matched control; “super p53” : mice with two extra copies of p53].

**Figure 3:**
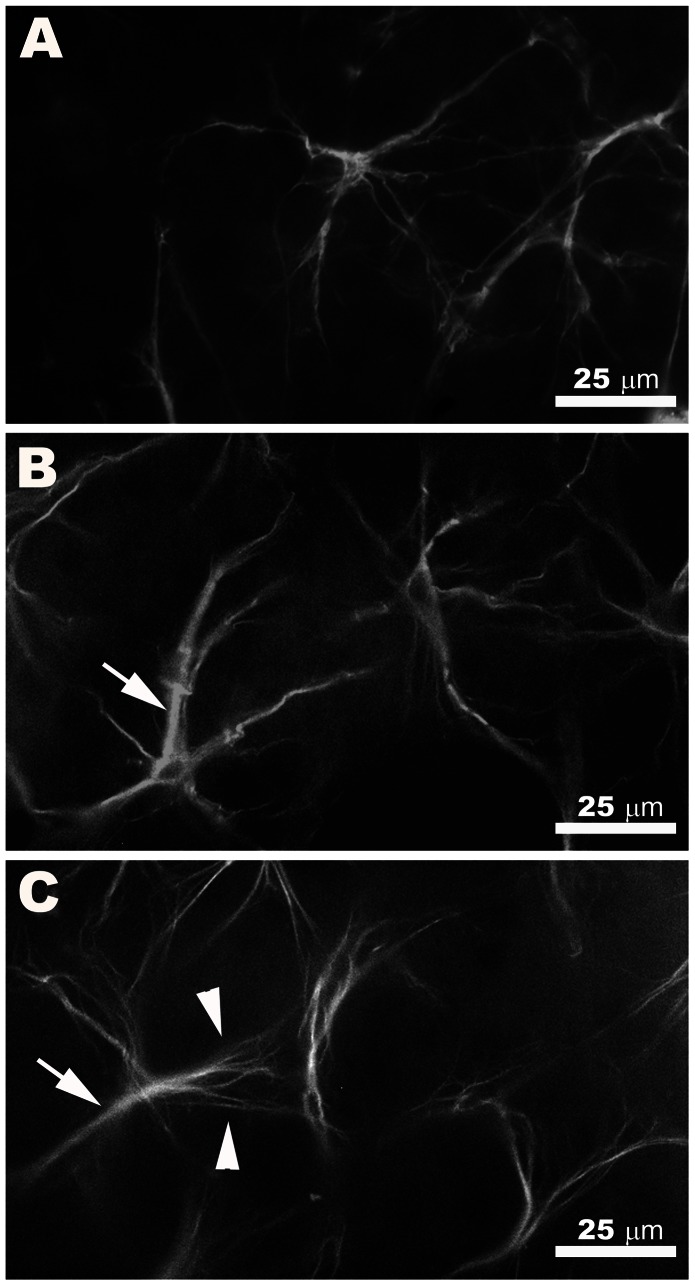
Morphological features of retinal astrocytes in WT and and “super p53” mice. GFAP immunofluorescence. A: WT. B–C: “super p53”. In WT and “super p53” eyes, astrocytes had a rounded body from which numerous primary and secondary processes extended. In “super p53” eyes the soma and the primary processes (arrow) had a more robust appearance than in WT. Secondary processes of astrocytes in “super p53” were more evident than WT and spread out like a fan (arrowhead). [WT: wild type p53 age-matched control; “super p53” : mice with two extra copies of p53].

In WT retinas, GFAP+ astrocytes were spaced regularly throughout the nerve-fibre retinal ganglion cell (RCG) layer as viewed from the surface, forming a homogeneous plexus (Fig. 2B) evenly distributed throughout the retina from the disc to the periphery. This plexus was composed mainly of stellate-shaped cells (Fig. 3A) that could easily be distinguished from each other (Fig. 2B). These cells had a rounded body from which numerous primary and secondary processes extended (Fig. 3A). Astrocyte processes reached other astrocytes or blood vessels and formed the astroglial plexus (Fig. 2B). Some areas of the retina showed a faint GFAP immunoreactivity (IR) in Müller cells which appeared as punctuate structures between the astrocytes and their radiating processes (Fig. 2B).

#### 1.2. super-p53 mice

**Figure 4:**
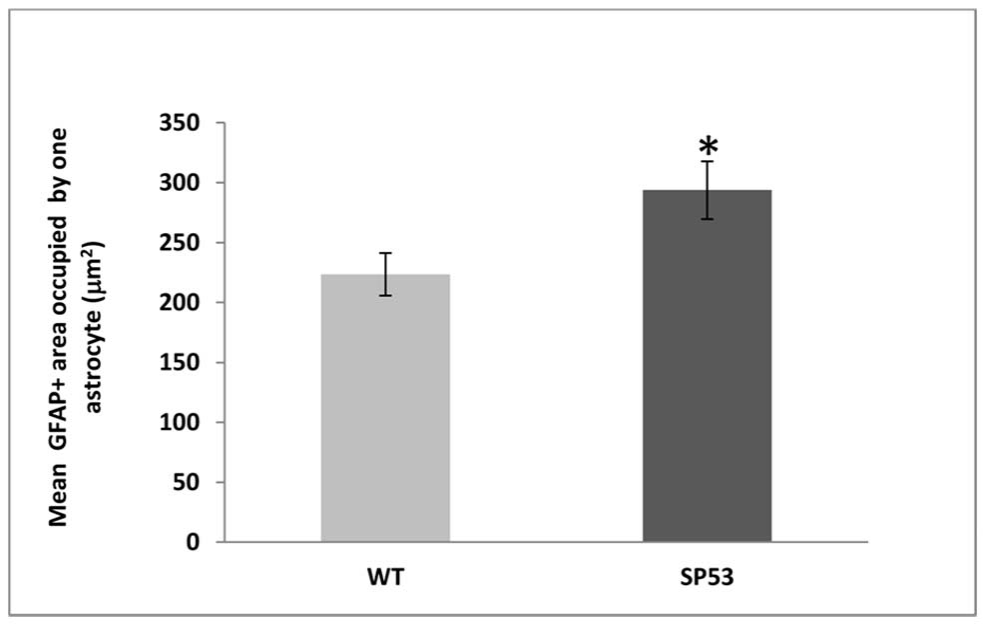
Mean GFAP+ area occupied by one astrocyte. In “super p53” retinas the mean GFAP+ area occupied by one astrocyte was significantly higher (*P<0.05; Student’s t-test) than in WT retinas. Columns represent mean ± SDM of GFAP+ area. [WT: wild type p53 age-matched control; “super p53” : mice with two extra copies of p53].

Like WT, astrocytes in “super p53” eyes formed a homogeneous plexus of star-shaped cells evenly distributed throughout the retina from the disc to the periphery. At first sight, the astrocyte plexus in “super p53” eyes appeared to be denser than in WT (Fig. 2). The analysis of the tissues at higher magnification, looking for morphological features that might contribute to this impression, revealed that the soma and primary processes of astrocytes in “super p53” retinas were apparently more robust and the secondary processes were more evident than in WT and spread out like a fan (Fig. 3). We calculated the mean area GFAP+ occupied by one astrocyte in order to ascertain whether this parameter could at least partly explain the subjective impression that in “super p53” retinas the astrocytes were more robust and the astroglial plexus denser than in WT. The analysis of these data revealed that the mean GFAP+ area occupied by one astrocyte in “super p53” eyes was significantly higher (p<0.05; Student’s t-test) (Fig. 4) than in the WT.

No differences in GFAP-IR in Müller cells were observed with respect to the WT.

#### 1.3. Astrocyte number

**Figure 5:**
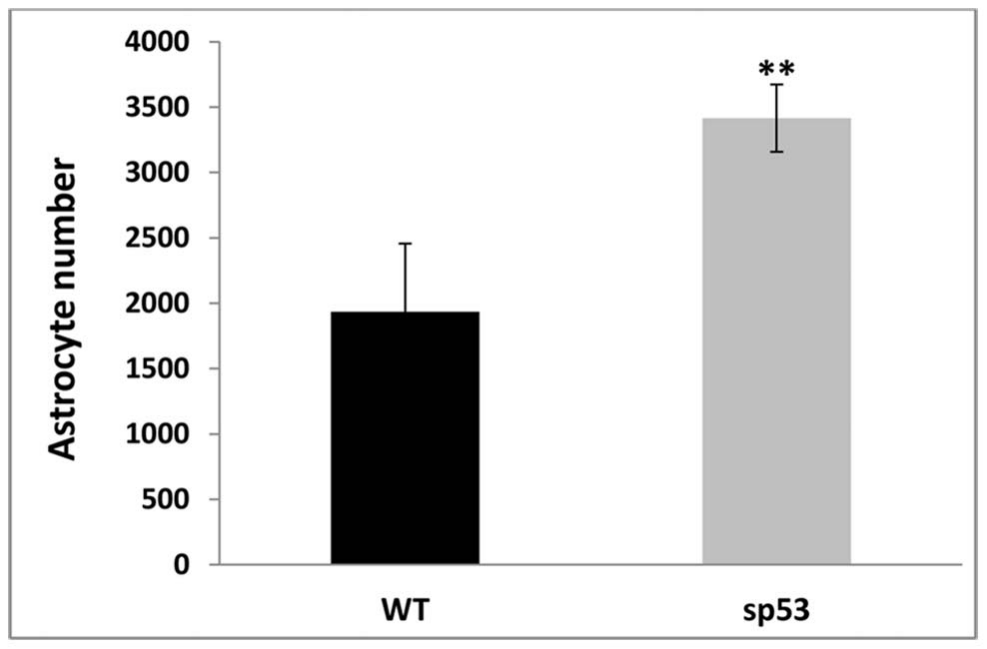
Astrocyte quantification in the entire retinal whole-mount. Total number of astrocytes in “super p53” retinas were significantly more numerous than in WT retinas (**p<0.01; Student’s t-test). Columns represent the mean number ± SDM of GFAP+ astrocytes [WT: wild type p53 age-matched control; “super p53” : mice with two extra copies of p53].

Another feature that could account for the denser appearance of the astrocyte plexus in “super p53” retinas was that the astrocytes were significantly more numerous (3414.80±258.00) than in the WT (1933.00±522.21) (p<0.01; Student’s t-test) (Fig. 5). In the analysis made by concentric zones, “super p53” animals had significantly more astrocytes than WT in the intermediate (70.76±10,69 vs. 53.38±8.51, respectively) and peripheral zones (71.11±12,11 vs. 41.19±12.91, respectively) (p<0.05, Student’s t-test in both instances).

## Discussion

Much of the early data reported on p53 were gathered using techniques that, in general, lack cell resolution, such as PCR, Western blot, and ELISA. Moreover, many of the initial studies linking p53 with nervous-system injury and degeneration somewhat overlooked the involvement of glia, and thus arrived at neuron-centred conclusions [10], [43]–[49]. To the best of our knowledge, this is the first study available to show the morphological features, distribution, and number of retinal astrocytes in “super p53” mice retina.

In the “super p53” mice retina, the supernumerary p53 gene was closely related to a significantly increased population of retinal astroglia when the quantification included the whole retina. This significant increase in astrocyte number was also detected in the intermediate and peripheral zones of the retina. In “super p53” the central zone, there was an area, near the optic-nerve margins, where the large amount of astrocytes made it difficult to discern cells individually, thus precluding quantification. This could be the reason why no significant differences in astrocyte number in the central zone were found between the two study groups.

The significant increase in the mean GFAP+ area occupied by one astrocyte in “super p53” eyes in comparison with WT, could explain our subjective impression that, in “super p53” mice retina, astrocytes were more robust and formed a denser astroglial network than in the WT mice. These features are exhibited by reactive astrocytes [50]–[53]. Studies of neurological disorders indicate that reactive gliosis may have either a positive or negative effect on neuronal function and maintenance [52]. The task of reactive astrocytes is assumed to be that of protecting the neurons by producing neurotrophic factors, increasing the expression of antioxidant enzymes, augmenting the transport and production of glucose [54], [55], regulating the removal of harmful substances produced by damaged neurons and promoting neuronal growth [56], [57], among others. Notably, these reactive gliosis-like changes of astrocytes in “super p53” retinas did not run in parallel with an increment in GFAP-immunoreactivity (IR) in Müller cells. By contrast, Ueki et al [58] analysed GFAP expression in retinal Müller cells in mouse Trp53−/− and found greater expression of this marker of reactive gliosis.

It has been proposed that an increase in p53 functionality, while beneficial to prevent tumour development, can be detrimental to long-term viability because it may accelerate the ageing process [59]. In human [60], [61] and rat [62], [63] retina, ageing is associated with a progressive decline in astrocyte number. The significant increase in the number of retinal astrocytes in the “super p53” group reinforces the idea that these mice lack signs of premature ageing. This may be related to the normal regulation of the supernumerary p53 animals [37].

The “super p53” retinas showed more astrocytes, which also had more evident secondary processes to interact with neighbouring neuronal cells. This could be a factor to increase the neuro-supporting role of astrocytes. In the human retina, astroglial processes join together by means of desmosomes [64], [65] and gap junctions [65] to form a mesh that reinforces the capillary network and supports the neurons present in the glial network. These kinds of junctions between processes have also been reported in rats [66] as well in other animal species [67], [68]. It is known that astrocytes play a decisive role in the metabolism of neurotransmitters and CO_2_. Moreover, ions, most sugars, amino acids, nucleotides, vitamins, hormones, and cyclic AMC pass through gap junctions. Apart from coordinating the metabolic activity of cell populations, gap junctions may also participate in electrical activities or amplify the consequences of signal transduction [69]. Recently another kind of cell-cell communication, i.e. tunneling-nanotubes (TNTs), has been described [70]. TNTs are thin membranous extensions that form channels between cells for intercellular communication and trafficking and are found in numerous cell types, including neurons and astrocytes [71], [72]. The p53 gene is involved in TNT development. Cell insults activate p53 and induce M-Sec overexpression, which can trigger F-actin polymerization and contribute to TNT development from the initiating cell membrane [73], [74]. The apparent increment of astrocyte secondary processes found in this study could mean an increase of their gap junctions and TNTs and, consequently, an improvement in cell-cell interactions and neural function.

There is clear evidence for a role of p53 in the regulation of oxidative stress [75]. Although the apoptotic activity of p53 is mediated, at least partly, by rising ROS levels [76], [77], a number of studies have shown a survival function for p53 in lowering intracellular ROS levels, involving the activity of p53-inducible genes such as TIGAR, sestrins [78], [79], aldehyde dehydrogenase-4 (ALDH4) [80], and others [81]. Most notably, this antioxidant function of p53 is important in the absence of acute stress, preventing the accumulation of DNA damage on a day-to-day basis [24]. An increased p53 gene dosage confers the retina with noticeable resistance to OS, presumably by boosting antioxidant activity and opening anti-apoptotic pathways [82]. We have recently reported an increase in the total antioxidant activity in the optic nerve and retina of “super p53” with respect to WT animals [83], [84]. This higher antioxidant activity could indicate that p53 may modulate OS-derived retinal damage. The higher number of astrocytes found in the retinas of “super p53” mice in the present work could be responsible for the augmented antioxidant capacity observed [83], [84].

The most noteworthy finding of this study is the significant increase in the retinal astrocyte population of “super p53” mice. This increase might improve the resistance of the retinal cells against ROS and its downstream signalling pathways. These findings could be the starting point to develop future treatments for those diseases such as diabetic retinopathy, glaucoma, or ARMD, the pathogenesis of which involves oxidative stress.
